# Effects of GA_3_ Pregerminative Treatment on *Gentiana lutea* L. var. *aurantiaca* Germination and Seedlings Morphology

**DOI:** 10.1155/2014/751279

**Published:** 2014-06-29

**Authors:** Óscar González-López, Pedro A. Casquero

**Affiliations:** ^1^Research Group of Engineering and Sustainable Agriculture, Natural Resources Institute, University of León, Avenue Portugal 41, 24071 León, Spain; ^2^Research Group of Engineering and Sustainable Agriculture, Department of Agrarian Engineering and Sciences, University of León, Avenue Portugal 41, 24071 León, Spain

## Abstract

*Gentiana lutea* L. is widely used in bitter beverages and in medicine; *Gentianae Radix* is the pharmaceutical name of the root of *G. lutea*. These uses have generated a high demand. The wild populations of *Gentiana lutea* var. *aurantiaca* (M. Laínz) M. Laínz have been decimated; it is necessary to establish guidelines for its cultivation. Gentian as most alpine species has dormant seeds. Dormancy can be removed by cold and by means of a gibberellic acid (GA_3_) treatment. However, cold treatments produce low germination percentages and GA_3_ treatments may produce off-type seedlings. So, the objective was to determine, for the first time, the presowing treatments that allow high germination rate and good seedling morphology. The best pregerminative doses of GA_3_ to break seed dormancy were 100, 500, and 1000 ppm, while the best doses to optimize the seedling habit were 50 and 100 ppm. This study provides, for the first time, a 100 ppm GA_3_ dose that led to a high germination rate and good seedling morphology, as the starting point for gentian regular cultivation.

## 1. Introduction

*Gentiana lutea* L. (*Gentianaceae*) is a herbaceous perennial plant native to the mountains of central and southern Europe [1, 2]. Gentian roots are widely used in bitter beverages, in food products, and also in traditional medicine to stimulate the appetite and improve digestion [3, 4]. These uses have generated a great demand, so that more than 1500 tons of gentian root is produced from 6000 tons of wild stocks every year around the world [5]. Due to the slow growth of this species, the wild populations of* G. lutea* have been decimated and are now close to their disappearance and/or are difficult to revive. The increasing demand has provoked alarm about the extinction of the species, and for this reason gentian is being protected throughout Europe by law.

In the Northwest part of Iberian Península (Western Cantabric Mountains), gentian root has been used as a tonic for the stomach and to restore appetite [6]. In this area* Gentiana lutea* L. flowers show a colour ranging from orange to almost red when compared to yellow flowers of* G. lutea *L. subsp.* lutea. *These populations have been classified as* Gentiana lutea* L. var.* aurantiaca* [7]. In the León province some populations of* aurantiaca* variety populations show signs of isolation with low level of genetic diversity [8] which indicates reduced reproductive fitness and elevated extinction risks [9].

The main economic sources in European mountain areas include mining (a decreasing activity), ranching (in crisis), and tourism. Collection of gentian roots has traditionally been a supplement to the family income. Due to the current economic crisis, people have started to collect gentian again in a furtive way for an extra income. Considering the harsh climate conditions of the mountain regions, where both horticulture and fruit growths are difficult to carry out, the cultivation of gentian is an alternative economic activity for large unused lands with agro climatic characteristics suitable for its growth.

*G. lutea*, as most alpine species, has dormant seeds, exhibiting mainly physiological dormancy [10, 11]. This dormancy can be removed by cold treatment [12, 13], by cold stratification [14], and by GA_3_ treatment. GA_3_, alone or together with other chemical or physical treatments, is widely used to remove seed dormancy [15] or to improve the development of the seedlings, although diverse results have been obtained [16–19]. Related to this, a study with* G. lutea* seeds was carried out on germination rates in Petri dishes using GA_3_ and a stratification treatment [20]. However, some of these treatments, such as gibberellic acid, may produce weak and off-type seedlings in* G. lutea* [21].

Therefore, this study aims to establish which GA_3_ dose, applied as pregerminative treatment, improves the germination rate without affecting the development of seedlings, as the first step for gentian cultivation.

## 2. Materials and Methods

### 2.1. General Seed Trials

**Figure 1:**
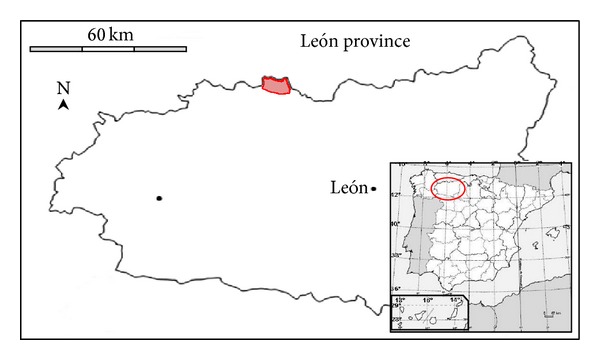
Map showing the location of sampled population of* Gentiana lutea* L. var.* aurantiaca*.

Seeds of* Gentiana lutea* L. var.* aurantiaca *from population located in the western part of the Cantabrian mountains (Spain) were collected (Figure 1). Seeds from mature capsules containing well-developed ripe seeds were taken from 10 randomly selected plants. After this, the seeds were manually cleaned, removing any damaged, empty, or visually malformed seeds, kept in paper bags, and then stored in a fridge at 4°C inside a dark glass bottle with silica gel.

**Figure 2:**
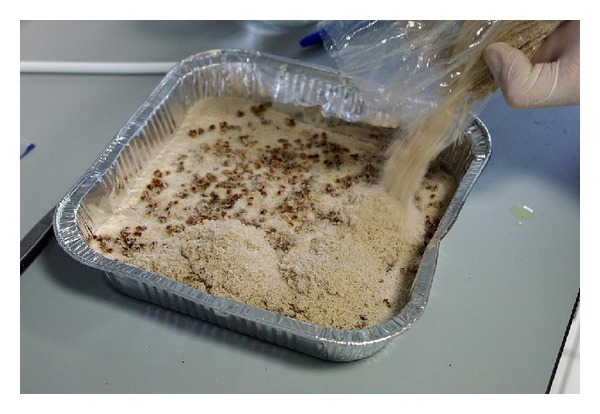
Addition of silica sand over gentian seeds for stratification treatment.

*Stratification Treatment.* Seeds were stratified for 3 months over a silica sand base (Ø 0.3–0.9 mm). Both tray and silica sand were sterilized by autoclaving at 1.0 kg cm^−2^ and 120°C for 1 h, on each of three consecutive days. After placing the seeds well distributed over the tray surface, another layer (1 cm in height) of silica sand was added covering the seeds (Figure 2). Finally, the sand was moistened with distilled water, avoiding saturation and placing the tray inside the refrigerator at 4°C ± 1°C. The tray was checked every week during the next 90 days in order to control the sand humidity. Stratified seeds were cleaned using a metallic sieve with Ø 1.5 mm.

*GA*_*3*_ *Treatment*. Seeds were soaked at 25°C for 24 hours in GA_3_ (Sigma-Aldrich) water solution (50, 100, 500, and 1000 ppm) and were cleaned with distilled water to remove GA_3_ residues before sowing.

### 2.2. Petri Dishes Germination Assay

**Figure 3:**
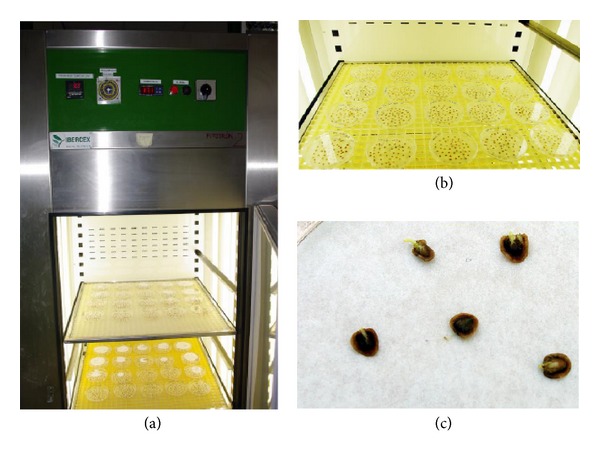
Petri dishes germination assay of* G. lutea* L. var.* aurantiaca*: (a) growth chamber; (b) Petri dishes distribution; (c) germinated seeds.

Two treatments were applied to the gentian seeds: cold treatment (stratified or not) and GA_3_ dose (0, 50, 100, 500 and 1000 ppm). Five replicates of 100 seeds in each treatment were tested for germination on top of a filter paper sheet with 4 ml distilled water in 9 cm plastic Petri dishes (Figure 3(b)). Filter papers were rewetted with distilled water as required. Dishes were checked twice a week during a 90-day test period and germinated seeds were counted and removed. It was established that seeds were germinated when the length of the radicle exceeded the seed coat with 1 mm (Figure 3(c)). The conditions during this time were in a growth chamber (Figure 3(a)) with a constant temperature of 15°C and a 16 h light photoperiod (provided by cool white fluorescent tubes with an irradiance of 35 *μ*mol*·*m^−2^ *·*s^−1^). At the end of the incubation period (90 days), the final germination percentage and the mean germination time (MGT) were calculated according to Ellis and Roberts [22].

### 2.3. Forest Trays Assay

Five replicates of 100 seeds each were tested with five GA_3_ dose (0, 50, 100, 500 and 1000 ppm). All seeds were stratified as specified in Section 2.1. The forestry seed trays had a volume of 200 cm^3^ and a height of 15 cm. A commercial substrate, n° 28 from Pindstrup Mosebrug Sae, was used. This substrate has a mix of 70% white peat and 30% black peat (pH 5.5–6), with similar conditions to those of the mountain soils. Pesticide seed treatment (Tiram 80% WP) was applied according to Valenciano et al. [23] to control the development of phytopathogenic fungi [24]. Every lot of 100 seeds was sowed in a unique tray and every cell was covered with 15 cm^3^ of sterilized silica sand, which is equivalent to 1 cm in height.

180 days after sowing, the measurement of the plants was carried out. Once the development of the plants was observed, two different characteristics were measured: stem length, that is, the length of the stem (mm) from the substrate level to the apex of the primary stem, and leaf length, the length (mm) of the most developed leaf in the third pair of leaves starting from the base. Both measurements were performed in 20 random seedlings per replication.

### 2.4. Statistical Analysis

The values of final germination percentages were arcsine transformed (untransformed data appears in the figures). In each experiment, the data was analyzed using the general linear model (GLM procedure) and whenever the analyses showed significance least significant differences (LSD) were computed at the 0.05 probability level to compare means. All analyses were performed using the SAS version 9.1.2 software (SAS Institute Inc., 2004, Cary, NC, USA).

## 3. Results and Discussion

### 3.1. Germination

**Figure 4:**
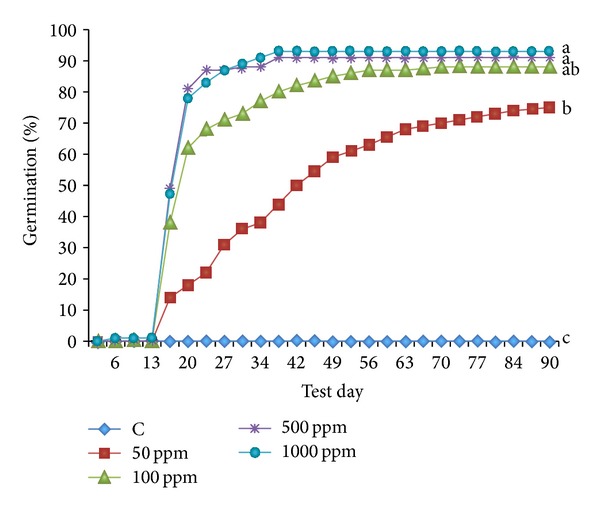
Germination curves of* Gentiana lutea *var.* aurantiaca *seeds using different doses of GA_3_ (50, 100, 500, and 1000 ppm) and control (C).

According to analysis of variance, stratified seeds showed higher germination rates than nonstratified seeds for each tested GA_3_ dose. There were no interactions between the sources of variation used (cold treatments and GA_3_ treatments). The GA_3_ treatment had an effect on germination time so a generalized early germination was observed when 500 and 1000 ppm doses were used (Figure 4). In these cases, after 21 days, most of the seeds were germinated. Medium germination time (MGT) for these doses was 19.82 ± 0.475 and 20.34 ± 0.545, respectively. Regarding 100 ppm, a quick germination was also observed, but not as strong (MGT = 23.77 ± 1.111), and newly germinated seeds were observed until the 56th day. Finally, the 50 ppm dose meant a good final germination rate, but the number of germinated seeds increased until the 90th day at a gradual rate (MGT = 39.16 ± 2.186).

**Figure 5:**
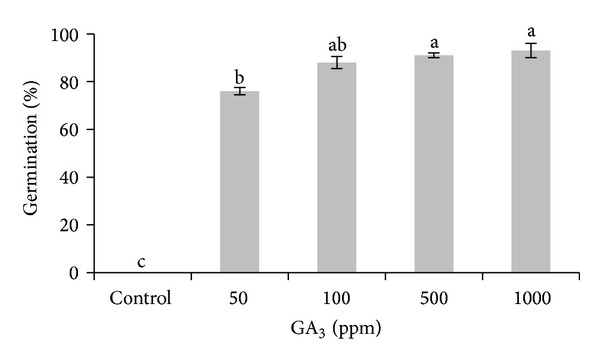
Germination percentages of* Gentiana lutea *var.* aurantiaca *seeds using different GA_3_ doses (50, 100, 500, and 1000 ppm) and control at the end of the test of 90 days of germination. Bars with different letters are significantly different (*P* = 0.05) according to LSD.

At the end of the 90 days of the germination test, none of the sterilized water pretreated seeds were germinated. All GA_3_ treatments yielded significantly higher germination percentages compared to the control. The greater the hormonal dose used, the greater the percentage of germination obtained. Seeds pretreated with 1000 ppm of GA_3_ resulted in the highest germination percentage (93%), not significantly different compared to the 100 ppm (91%) or 500 ppm (88%) doses, but significantly different to the 50 ppm dose (76%) (Figure 5).

The results obtained in Petri dishes germination test are in accordance with those obtained by Pérez-García et al. [20], which verify the high degree of success in the elimination of seed dormancy using GA_3_, this effect being greater with high doses (Figure 5). The highest GA_3_ doses (500 and 1000 ppm) provided a quick and grouped germination. The effect of the GA_3_ dose of 100 ppm was that the germination was slower during the first part of the test compared to higher GA_3_ doses, but MGT and germination were similar for all three doses.

### 3.2. Seedlings Development

**Figure 6:**
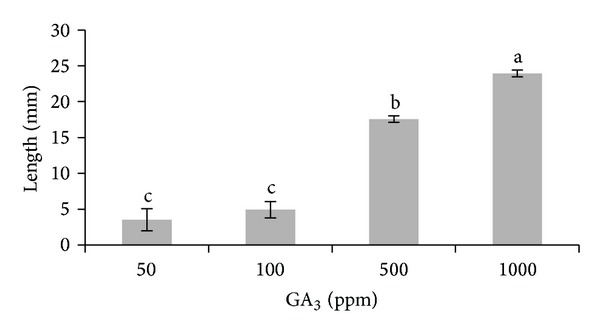
Stem length of* Gentiana lutea *var.* aurantiaca *seedlings using different GA_3_ doses (50, 100, 500, and 1000 ppm) on the 90th day after sowing. Bars with different letters are significantly different (*P* = 0.05) according to LSD.

Analysis of variance shows significant differences between GA_3_ treatments. Stem length was greater when the GA_3_ dose used was higher. The dose of 1000 ppm provided the longest (23.93 mm) stem, being significantly different from the others, as well as the 500 ppm dose (17.58 mm). Significant differences were not found between the remaining doses of 50 ppm and 100 ppm, both providing the shortest stems of all the treatments (4.94 mm and 3.53 mm, resp.) (Figure 6).

**Figure 7:**
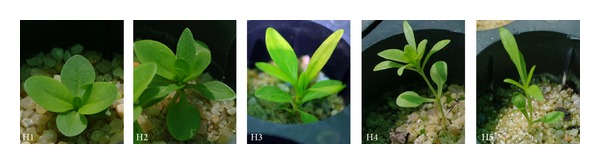
Growth habits (H1–H5) of* Gentiana lutea *var.* aurantiaca *seedlings on the 180th day after sowing treated seeds with GA_3_ (50, 100, 500, and 1000 ppm).

Depending on the GA_3_ dose, gentian seedlings showed different growth habits (Figure 7). H1: the aerial part of the plant is formed by a basal rosette of well-developed leaves and the stem is very short. H2: the aerial part of the plant is formed by a basal rosette of well-developed leaves; the stem is formed and strong. H3: the leaves are elongated; they are arranged along a stem formed with short internodes. H4: small leaves are arranged on a very long and weak stem: long internodes; light yellow-green color. H5: poorly developed and large leaves: filiform appearance; long internodes; light yellow-green color.

**Figure 8:**
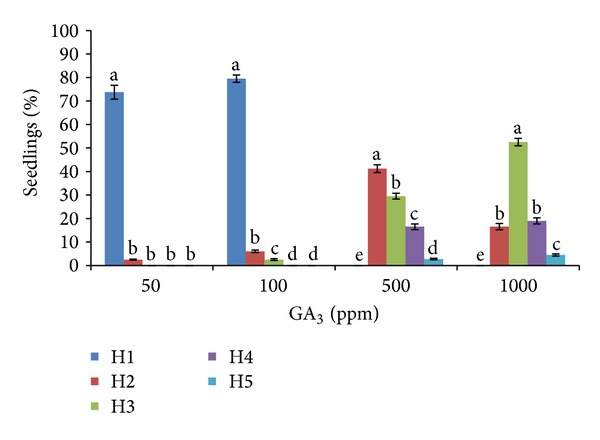
Percentage of* Gentiana lutea *var.* aurantiaca *seedlings showing different growth habits for each of the GA_3 _doses (50, 100, 500, and 1000 ppm). Bars with different letters for each GA_3_ doses are significantly different (*P* = 0.05) according to LSD.

Considering these five growth habits the number of plants showing each of the habits was counted according to the different GA_3_ doses (Figure 8). The habit shown by the seedlings at low doses was similar, H1 being the predominant. Meanwhile, for high GA_3_ doses, 500 and 1000 ppm, the predominant growth habit was H3, and also H4 was abundant. In both, 500 ppm and 1000 ppm, none of the seedlings showed the H1 habit, which is considered the best for the gentian seedlings.

The negative effect on the development of the seedlings was observed only at 500 and 1000 ppm doses, whereas a high percentage of seedlings showed off-type growth habits with long stems. Using these doses, seedlings were very weak, poorly developed, and with a threadlike appearance, therefore, more susceptible to low temperatures, insect attacks, physical breakage, or drying out. While at low doses (50 ppm and 100 ppm) the stems were very short and the predominant seedlings morphology was very similar to that observed in nature, where the seedlings are formed by a basal rosette, like other perennial mountainous plants, protecting themselves from the hard conditions of the mountain climate.

## 4. Conclusion

The best GA_3_ pregerminative doses to break seed dormancy of* Gentiana lutea* L. var.* aurantiaca *were 100, 500, and 1000 ppm. Doses of 500 and 1000 ppm GA_3_ not only allow obtaining a high and uniform germination, but also produce high percentages of weak and off-type seedlings. The best doses to optimize seedling habit were 50 and 100 ppm. This study provides, for the first time, a 100 ppm GA_3_ doses that led to a high germination rate and good seedling morphology, as the starting point for gentian regular cultivation.
